# Treatment effect based on antimicrobial resistance in Staphylococcus aureus infections related to orthopedics

**DOI:** 10.1038/s41598-025-33687-z

**Published:** 2025-12-24

**Authors:** Fei Liu, Aijing Li, Linjie Tu, Yuqin Zhu, Qi Yao, Ping Yuan, Bing Ge

**Affiliations:** 1 Department of Emergency, The Fourth People’s Hospital of Guiyang, Guiyang, The People’s Republic of China; 2 Department of Critical Care Medicine, The Fourth People’s Hospital of Guiyang, Guiyang, The People’s Republic of China; 3 Clinical Laboratory, The Fourth People’s Hospital of Guiyang, Guiyang, The People’s Republic of China; 4 The Fourth People’s Hospital of Guiyang, Guiyang, The People’s Republic of China

**Keywords:** Staphylococcus aureus, Infections related to orthopedics, Antimicrobial resistance, Oxacillin, Multidrug resistance, Antimicrobials, Pathogens, Epidemiology

## Abstract

*Staphylococcus aureus* is a prevalent pathogen in infections related to orthopedics. Here, we report on the antimicrobial resistance and treatment with emphasis on both clinical and microbiological perspectives. We recruited 196 patients with *S. aureus* isolates. Antimicrobial resistance was described by resistance rates, and multidrug resistance (MDR) was classified according to international standards. Antimicrobial agents were classified as active, untested, or inactive according to the antimicrobial susceptibility tests. The treatment stage was divided into empirical and targeted stages, and effects were classified as first-, second-, or third-grade healing. Resistance rates ranged from 0.0 to 89.2%, and no resistance to vancomycin or linezolid was found. As the signal of methicillin resistance, the incidence of oxacillin resistance was 21.0%. MDR was identified in 84 (42.9%) isolates, not related to gender (*p* = 0.505) or age (*p* = 0.459). Cefuroxime and cefazolin were common untested agents, whereas clindamycin and levofloxacin were common active agents used in treatment. Out of the participants, 173 had first-grade healing, 16 had second-grade healing, and 7 had third-grade healing. Of the patients who received active and untested agents, the numbers of patients who had first-, second-, and third-grade healing were 74, 6, and 4 vs. 99, 10, and 3 (*p* = 0.709), respectively. Among the patients who received untested agents during empirical treatment, 20.9% (28/134) switched to active agents for treatment success. Antimicrobial resistance is a prevalent concern, especially methicillin resistance. Active agents represented by clindamycin and levofloxacin are valuable according to antimicrobial susceptibility tests and clinical practice.

## Introduction

*Staphylococcus aureus* is one of the most prevalent pathogens in infections related to orthopedics, and represents a key member of the ESKAPEE group^1^ (*Enterococcus faecium*, *S. aureus*, *Klebsiella pneumoniae*, *Acinetobacter baumannii*^2^, *Pseudomonas aeruginosa*, *Enterobacter spp.*, and *Escherichia coli*^3^, which encompasses the most problematic multidrug-resistant pathogens in healthcare settings. Infections related to orthopedics included community-acquired infections related to bones and joints^4^, such as osteomyelitis and septic arthritis, and hospital-acquired infections, represented by the skin and soft tissues infections after orthopedic surgeries^5^.

From a microbiological perspective, S. aureus exhibits diverse resistance mechanisms, including beta-lactamase production, altered penicillin-binding proteins (PBP2a in MRSA), and efflux pumps, which complicate treatment strategies in infections related to orthopedics where biofilm formation often occurs^1^,^4^.

During the treatment of *S. aureus*-related infections related to orthopedics, many antimicrobial agents may be used, depending on the antimicrobial resistance to methicillin. Although first- and second-generation cephalosporins are traditionally considered reliably active against methicillin-susceptible *S. aureus* (MSSA)^6,7^, emerging reports describe reduced susceptibility or borderline resistance - particularly to cefazolin^8,9^. Understanding local cephalosporin susceptibility patterns, even for MSSA, remains clinically relevant in deep-seated orthopedic-related infections. For methicillin-resistant *S. aureus* (MRSA), vancomycin^10,11^ and daptomycin^12,13^ are the first options. Moreover, alternative therapies, represented by linezolid^14^, are proven effective. Recently, resistance rates to common antimicrobial agents such as methicillin have increased^15^; even for high-end antimicrobial agents such as vancomycin, resistance has been occasionally reported in recent years^16–19^. In this situation, antimicrobial susceptibility tests help guide targeted treatment^10,20^, and a large specimen size of antimicrobial susceptibility tests helps us further understand the situation of antimicrobial resistance and guides empirical treatment.

Several questions remain unanswered. First, the basic situation of antimicrobial resistance of *S. aureus* causing infections related to orthopedics is unknown. Second, the common antimicrobial agents and related treatment effects are pending in recent years. Finally, the value of antimicrobial agents according to MSSA or MRSA and local antimicrobial resistance is unknown.

To understand *S. aureus*-related infections related to orthopedics in more depth, we reported pathogen antimicrobial resistance, treatment, and clinical outcomes from both clinical and microbiological perspectives. We also discussed the value of antimicrobial susceptibility tests in guiding treatment.

## Methods

### Study design

We retrospectively recruited patients with *S. aureus-*related infections related to orthopedics from 2021 to 2022 at Guiyang Fourth People’s Hospital (Guiyang, China), a 1,000-bed tertiary hospital and one of the largest orthopedics specialty centers in Guizhou Province, serving a catchment population of approximately 3.8 million residents.

### Inclusion and exclusion criteria

The inclusion criterion consisted of patients with *S. aureus* infections isolated from secretion cultures in the departments related to orthopedics. The exclusion criteria consisted of patients with infections acquired at other hospitals, those with incomplete data, patients with unknown strains, and clinically insignificant cultures. The diagnosis of infections related to orthopedics were independently assessed by two experienced clinicians.

### Microbiological diagnostics

Species identification and antimicrobial susceptibility testing were performed using the WALKWAY-96 system (Siemens, Germany), an automated system, following the manufacturer’s protocols. Clinical specimens were inoculated on appropriate media by experienced laboratory technicians and incubated for up to seven days. Minimum inhibitory concentrations were determined using the broth microdilution method according to Clinical and Laboratory Standards Institute (CLSI) M100 guidelines (31st edition, 2021)^21^. Molecular techniques for resistance gene detection were not routinely performed. Quality control was performed using S. aureus ATCC 29,213. MRSA was defined by oxacillin minimum inhibitory concentrations ≥ 4 µg/ml via broth microdilution interpreted per CLSI M100^21^.

### Antimicrobial susceptibility tests

Fifteen antimicrobial agents were tested and classified by pharmacological class: beta-lactams (penicillins, cephalosporins), glycopeptides (vancomycin), lipopeptides (daptomycin), oxazolidinones (linezolid), streptogramins (quinupristin/dalfopristin), fluoroquinolones, aminoglycosides, and folate pathway inhibitors.

### Antimicrobial resistance

The antimicrobial resistance was reported by resistance rates^21^. Multidrug-resistant (MDR) was defined as non-susceptibility to at least one agent in three or more antimicrobial categories; extensively drug-resistant (XDR) was defined as susceptibility to only one or two antimicrobial categories; and pan-drug-resistant (PDR) was defined as non-susceptibility to all agents in all antimicrobial categories^2,3,22^.

### Treatment

The treatment stage was divided into empirical and targeted treatment based on the reports of culture and antimicrobial susceptibility tests. An active antimicrobial agent indicates that the strain of bacteria was susceptible or intermediate (susceptible-dose-dependent). The presence of inactive antimicrobial agents indicates that the bacterial strain was resistant. Untested antimicrobial agents mean that antimicrobial susceptibility tests were not performed, and the antimicrobial agent was conventionally used to treat *S. aureus*-related infections related to orthopedics. Debridement surgery was also described.

The choice of antimicrobial agent and its dosage was at the discretion of the attending physician, following institutional guidelines and standard clinical practice. Combination therapy was not routinely employed as a first-line strategy for the majority of uncomplicated infections related to orthopedics in this cohort. The primary focus was on adequate surgical debridement and targeted monotherapy based on susceptibility results. The primary factor leading to a change in therapy was the result of the antimicrobial susceptibility tests, which indicated that the empirically used agent was inactive (“resistant”) against the patient’s isolate. Furthermore, when the treatment effect is not as expected, the treatment should be considered to be replaced.

The antimicrobial agents have a priority level from active, untested, to inactive. When an agent with a higher priority level was discussed in a stage, the antimicrobial agents with lower priority levels were ignored.

### Clinical outcome

The effect of treatment on infections was divided into first, second, and third grades. The discharge outcome was dichotomized as dead or alive. The clinical outcome was determined on the day of discharge from the hospital, where patients with infections were treated.

### Data

We also collected demographic characteristics, basic health information, surgical histories, and infection-related information.

### Statistical analysis

We presented categorical variables as frequencies and percentages, and continuous variables as means and standard deviations. We performed statistical analyses using the R Programming Language, version 4.0.2. The characteristics of the groups were compared using contingency table analysis for categorical variables. Additional analyses included chi-square tests for resistance patterns by demographic variables. Statistical significance was set at *P* < 0.05.

## Results

### Participants

**Fig. 1:**
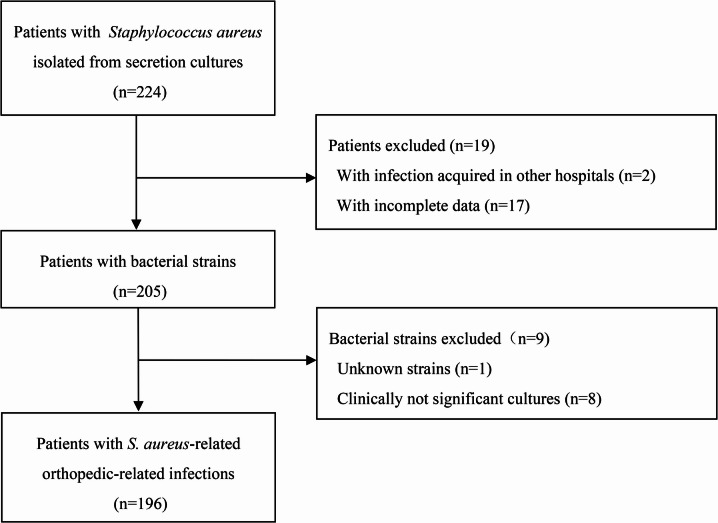
Flow chart of subject selection.

We recruited 196 patients with *S. aureus*-related infections related to orthopedics (Fig. 1). For all patients, the mean age was 38.7 ± 18.9 years (ranging from 1 to 87 years), 49 (25.0%) were female, and the main diagnosis was trauma in 173 (88.3%) cases (Table 1).

**Table 1:** Clinical characteristics of the cases.

Variables	Overall
	*n* = 196
Age (y), mean ± SD	38.7 ± 18.9
Female, n (%)	49 (25.0)
Main diagnosis, n (%)	
Trauma	173 (88.3)
Other diseases	23 (11.7)
Grades of incision^,^ n (%)	
I	27 (13.8)
II	12 (6.1)
III	148 (75.5)
Without operations	9 (4.6)
Type of infection, n (%)	
Community-acquired^a^	178 (90.8)
Hospital-acquired^b^	18 (9.2)
Location of infection, n (%)	
Upper limbs	112 (57.1)
Lower limbs	75 (38.3)
Trunk	8 (4.1)
Multiple parts	1 (0.5)
Chronic diseases^c^,^,^ n (%)	30 (15.3)
Severe complications^d,^ n (%)	3 (1.5)
Debridement, n(%)	163 (83.2)
Grades of healing incision^,^ n (%)	
First	173 (88.3)
Second	16 (8.2)
Third	7 (3.6)

^a^Post-traumatic wound infections, including skin and soft tissue infections related to fractures (*n* = 158), septic arthritis (*n* = 9), and osteomyelitis (*n* = 6); diabetic foot infections (*n* = 3); and primary infections (unspecified origin; *n* = 2).

^b^Post-traumatic wound infections without prosthetic involvement (*n* = 13), prosthetic material-related infections (*n* = 4) and diabetic foot infection (*n* = 1).

^c^Hypertension in 20 cases, diabetes mellitus in 16 cases, renal insufficiency in one case, and coronary heart disease in one case. Seven cases had more than one disease.

^d^Including one osteomyelitis and two sepsis.

### Antimicrobial resistance of pathogens

Fifteen antimicrobial agents were used in the antimicrobial susceptibility tests. Resistance rates ranged from 0.0 to 89.2%, with vancomycin and linezolid being the lowest, and the penicillin resistance rate was the highest. As the signal of methicillin resistance, the incidence of oxacillin resistance was 21.0%. Moreover, the rates of resistance to rifampin (0.5%), quinupristin-dalfopristin (2.6%), and daptomycin (3.6%) were less than 5%. Resistance rates to moxifloxacin (9.7%), levofloxacin (11.7%), trimethoprim-sulfamethoxazole (11.7%), ciprofloxacin (13.3%), and gentamicin (15.8%) were lower than 20% (Table 2).

**Table 2:** The antimicrobial agents used in the antimicrobial susceptibility tests and the results.

Antimicrobial agents	Tested	Resistant, *n* (%)	Intermediate, *n* (%)	Susceptible, *n* (%)
Vancomycin	196	0 (0.0)	2 (1.0)	194 (99.0)
Levofloxacin	196	23 (11.7)	5 (2.6)	168 (85.7)
Moxifloxacin	196	19 (9.7)	4 (2.0)	173 (88.3)
Gentamicin	196	31 (15.8)	1 (0.5)	164 (83.7)
Quinupristin-dalfopristin	196	5 (2.6)	2 (1.0)	189 (96.4)
Rifampin	196	1 (0.5)	1 (0.5)	194 (99.0)
Trimethoprim-sulfamethoxazole	196	23 (11.7)	0 (0.0)	173 (88.3)
Erythromycin	196	128 (65.3)	4 (2.0)	64 (32.7)
Ciprofloxacin	196	26 (13.3)	2 (1.0)	168 (85.7)
Clindamycin	195	43 (22.1)	2 (1.0)	150 (76.9)
Linezolid	195	0 (0.0)	0 (0.0)	195 (100.0)
Oxacillin	195	41 (21.0)	0 (0.0)	154 (79.0)
Penicillin	194	173 (89.2)	0 (0.0)	21 (10.8)
Daptomycin	194	7 (3.6)	0 (0.0)	187 (96.4)
Tetracycline	191	55 (28.8)	5 (2.6)	131 (68.6)

### Antimicrobial resistance patterns by patient demographics

We analyzed antimicrobial resistance patterns stratified by gender and age groups (< 18, 18–65, > 65 years). MDR was observed in 84/196 (42.9%) isolates, with no significant difference by gender (female: 46.9% vs. male: 41.5%; *p* = 0.505) or age group (*p* = 0.459). No XDR or PDR strains were detected. (Table 3).

**Table 3:** The antibiotic resistance patterns for the bacterial strains according to the gender and the age of the patients.

	Strains	MDR, *n* (%)	XDR, *n* (%)	PDR, *n* (%)
Gender				
Female	49	23 (46.9)	0 (0.0)	0 (0.0)
Male	147	61 (41.5)	0 (0.0)	0 (0.0)
Age, years				
< 18	29	14 (48.3)	0 (0.0)	0 (0.0)
18–65	152	62 (40.8)	0 (0.0)	0 (0.0)
> 65	15	8 (53.3)	0 (0.0)	0 (0.0)

### Treatment

Cefuroxime, cefazolin, clindamycin, and levofloxacin were administered to 79, 35, 31, and 22 patients in the empirical treatment stage, respectively. Cefuroxime, cefazolin, clindamycin, and levofloxacin were administered to 62, 26, 34, and 32 patients in the targeted treatment stage, respectively. Furthermore, 163 patients received debridement surgeries.

### Clinical outcome

Of all patients included, 173 had first-grade healing, 16 had second-grade healing, and 7 had third-grade healing. All patients were discharged alive (Table 1).

### Clinical outcome based on MRSA/MSSA

Of the 41 patients with MRSA, 37 (90.2%) had first-grade healing, 3 (7.3%) had second-grade healing, and 1 (2.4%) had third-grade healing; of the 155 patients with MSSA, 136 (87.7%) had first-grade healing, 13 (8.4%) had second-grade healing, and 6 (3.9%) had third-grade healing (*p* = 0.881).

### The effect of active antimicrobial agents and untested antimicrobial agents

A total of 84 patients received active antimicrobial agents, including clindamycin (35 patients) and levofloxacin (36 patients); 74 patients had first-grade healing, 6 had second-grade healing, and 4 had third-grade healing. Additionally, 112 patients received untested antimicrobial agents, represented by cefuroxime (67 patients) and cefazolin (27 patients); 99 patients had first-grade healing, 10 had second-grade healing, and 3 had third-grade healing (*p* = 0.709).

Among the patients who received active antimicrobial agents, 51 received them since the empirical treatment stage, 48 had first-grade healing, and three had second-grade healing; 33 patients received them in the targeted treatment stage, 26 had first-grade healing, 3 had second-grade healing, and 4 had third-grade healing (*p* = 0.036).

Among the 134 patients who received untested antimicrobial agents during empirical treatment, 28 were switched to active antimicrobial agents for treatment success.

## Discussion

Our study integrates clinical and microbiological perspectives on *S. aureus* infections related to orthopedics. Antimicrobial resistance is prevalent in *S. aureus*-related infections related to orthopedics. As a signal of MRSA, the oxacillin resistance rate was 21.0%, which is lower than the average rate reported in some large-scale global and national surveillance studies^23^. This may reflect regional variations in MRSA prevalence or the specific characteristics of our orthopedics patient population and explains why first- or second-generation cephalosporins were used widely in our institute^8^. From a microbiological standpoint, the absence of vancomycin and linezolid resistance is reassuring, though the emergence of daptomycin non-susceptibility (3.6%) warrants monitoring given its importance in MRSA treatment. Considering their expenses and adverse effects, many antimicrobial agents with low resistance rates, including rifampin and levofloxacin, can be used as empirical treatments with continuous monitoring.

The classification of MDR/XDR patterns reveals that nearly half of isolates exhibit MDR, emphasizing the challenge of empirical therapy in orthopedic settings. The lack of demographic variation in resistance patterns suggests that resistance patterns did not significantly vary across demographic subgroups, suggesting that bacterial factors rather than host characteristics primarily drive resistance profiles in this cohort, and the universal infection control and stewardship approaches may be more effective than targeted strategies based on patient characteristics.

Cefuroxime, cefazolin, clindamycin, and levofloxacin were the primary empirical and targeted treatments. The treatment schedule was consistent with that used in previous studies on *S. aureus*-related infections^9,24^ or antimicrobial susceptibility tests. Moreover, 84.0% of the patients underwent debridement surgery. The clinical outcomes of patients with *S. aureus*-related infections related to orthopedics were acceptable. All patients survived, and 88.8% had first-grade healing. When comparing outcomes based on methicillin resistance, we did not observe a trend towards lower rates of first-grade healing in the MRSA group compared to the MSSA group. The possible reason could be that the inactive agents were replaced timely during hospitalization.

In the analysis of the value of antimicrobial susceptibility tests in the guidance of treatment, we compared the treatment effects of active and untested antimicrobial agents. Numerically, patients who received only the untested antimicrobial agents experienced better treatment outcomes. However, we found that 20.9% of patients who received untested antimicrobial agents, such as cefuroxime and cefazolin, experienced treatment failure and had to change to active antimicrobial agents, such as clindamycin and levofloxacin, for treatment success. In the analysis of the value of antimicrobial agents, we used the treatment effect as the study outcome and the antimicrobial agents as the exposure because the treatment effect is affected by bacteria^25^, treatment^26^, and immunity^27^. The bacteria were single, and immunocompromised patients were not found. And we thought that the analysis of treatment effect based on treatment was reliable on susceptibility testing significantly improves outcomes, supporting the value of laboratory-guided therapy. The superior performance of active agents in empirical treatment highlights the importance of local antibiogram data for informing initial therapy choices.

Overall, timely active antimicrobial agents should be administered, and antimicrobial susceptibility tests in local institutes are more effective in the guidance of treatment than the global experience for the following reasons. First, active antimicrobial agents did not work worse than untested antimicrobial agents. Second, one-fifth of patients who received untested antimicrobial agents as empirical treatment needed to receive active antimicrobial agents for treatment success. Finally, active antimicrobial agents as empirical treatment demonstrated superior efficacy than active antimicrobial agents as targeted treatment.

Considering that the active antimicrobial agents were consistent with the antimicrobial susceptibility tests and clinical practice, we found clindamycin and levofloxacin to be reliable antimicrobial agents for empirical treatment. Even after the report of antimicrobial susceptibility tests, the antimicrobial agents were shown to be inactive, and the rate of change of treatment schedules was still lower than that of the untested antimicrobial agents.

This study has several strengths. First, it integrates detailed microbiological data with comprehensive clinical treatment and outcome information, providing a holistic view of the management of *S. aureus* infections related to orthopedics. Second, the analysis is based on a substantial, well-defined patient cohort from a major orthopedic specialty center, ensuring the clinical relevance and representativeness of our findings for the regional population. Furthermore, we provided a practical analysis of the real-world value of antimicrobial susceptibility testing by comparing treatment outcomes between active and untested agents, and highlighting the significant proportion of patients requiring a switch to active drugs for success. Finally, the clear identification of locally effective agents like clindamycin and levofloxacin offers immediate, evidence-based guidance for empirical therapy in our setting, which is crucial for antimicrobial stewardship.

This study has inherent limitations. First, this was a retrospective study, and more evidence from clinical practice is necessary. Second, we did not include molecular epidemiological data (e.g., SCCmec or spa typing) or investigate phenotypes such as Small Colony Variants (SCVs), which could provide further insights into strain characteristics and persistence. These analyses were beyond the scope of this clinical outcomes study. Third, we did not discuss the value of oxacillin in clinical practice because only two patients received it as a targeted treatment. Finally, outcomes were assessed at discharge; data on outpatient therapy continuation and long-term outcomes were not captured.

## Conclusion

Antimicrobial resistance is prevalent in S. aureus causing infections related to orthopedics, with MDR present in about half of isolates. Microbiological diagnostics and local susceptibility patterns are essential for guiding effective therapy. Active antimicrobial agents represented by clindamycin and levofloxacin demonstrate consistent efficacy in both susceptibility testing and clinical practice. Integration of microbiological perspectives with clinical management improves patient outcomes in infections related to orthopedics.

## Data Availability

The datasets used and/or analysed during the current study are available from the corresponding author on reasonable request.

## Abbreviations

MSSA: Methicillin-susceptible S. aureus
MRSA: Methicillin-resistant S. aureus
MDR: Multidrug-resistant
XDR: Extensively drug-resistant
PDR: Pan-drug-resistant
